# Enhanced performance and stability in InGaZnO NIR phototransistors with alumina-infilled quantum dot solid

**DOI:** 10.1038/s41598-022-16636-y

**Published:** 2022-07-16

**Authors:** Yoon-Seo Kim, Hye-Jin Oh, Seungki Shin, Nuri Oh, Jin-Seong Park

**Affiliations:** Division of Materials Science and Engineering, Hanyang University, 222, Wangsimni-ro, Seongdong-gu, Seoul, 04763 Republic of Korea

**Keywords:** Engineering, Materials science, Optics and photonics

## Abstract

The optimized ALD infilling process for depositing Al_2_O_3_ in the vertical direction of PbS QDs enhances the photoresponsivity, relaxation rate and the air stability of PbS QDs hybrid IGZO NIR phototransistors. Infilled Al_2_O_3_, which is gradually deposited from the top of PbS QDs to the PbS/IGZO interface (1) passivates the trap sites up to the interface of PbS/IGZO without disturbing charge transfer and (2) prevents QDs deterioration caused by outside air. Therefore, an Al_2_O_3_ infilled PbS QD/IGZO hybrid phototransistor (AI-PTs) exhibited enhanced photoresponsivity from 96.4 A/W to 1.65 × 10^2^ A/W and a relaxation time decrease from 0.52 to 0.03 s under NIR light (880 nm) compared to hybrid phototransistors without Al_2_O_3_ (RF-PTs). In addition, AI-PTs also showed improved shelf stability over 4 months compared to RF-PTs. Finally, all devices we manufactured have the potential to be manufactured in an array, and this ALD technique is a means of fabricating robust QDs/metal oxide hybrids for optoelectronic devices.

## Introduction

Near infrared (NIR) photosensors have great potential in various electronic applications such as home appliances, mobile and healthcare devices, and automobiles due to the ability of NIR light to detect changes in the surrounding environment. For example, NIR can penetrate into human skin, allowing wearable electronic devices integrated with NIR sensors to monitor vital signs. For example, the difference in NIR absorption between oxygenated and deoxygenated hemoglobins indicates real-time information about blood oxygen saturation and heart rate^1,2^. Vein recognition sensors that can map veins using NIR absorption by red blood cells are also attractive applications of the NIR sensor arrays^3^. In addition, the NIR image sensors can provide good vision even in poor visibility conditions by measuring NIR reflected from objects in the dark or in extreme weather conditions^4^. Since the integration of the NIR photosensors with other electronics enables rich interactions among various entities, fast responsive and reliable NIR sensor arrays are required.

Metal oxide semiconductors are attractive candidates that enable fabrication of reliable optical sensor arrays based on their excellent properties, including high transparency, high mobility, low off current and low processing temperatures^5,6^. Of the oxide semiconductors we studied, the most commercialized indium-gallium-zinc oxide (IGZO)-based thin film transistors (TFTs) have high uniformity over a large area, low off current and low power consumption. However, due to the wide optical band gap of InGaZnO (> 3 eV, which is not suitable to absorb NIR light), a hybrid structure combined with NIR-active materials has been considered. Many researchers have attempted to detect NIR light by applying a light absorption layer such as quantum dots (QDs)^7^, perovskite^8^, nanoparticles^9^, or two-dimensional materials^10^. Among these NIR-absorbing materials, QDs have been widely investigated because of their wide tunability of absorption wavelengths and the fact that they can be mass produced through wet-chemical synthesis. Facile solution processibility using inkjet or spin-casting can also allow these QDs to be used in next-generation displays and image sensors. However, the increased surface area of QDs normally results in many surface trap-states. Those unstable states on the QD surface are inevitably generated by dangling bonds, atomic vacancies, and oxidative species, resulting in poor stability performances including short device operation lifetime, and easy degradation by oxygen and moisture under illumination. To overcome these issues, many studies have been reported such as the ligand exchange process^11^, core–shell structures^12^, and the application of a passivation layer^13^, but the need to improve the photostability of QDs remains.

QD-hybrid phototransistors should meet the requirements of high photoresponsivity as well as photostability. For high photoresponsivity, it is important that a large number of electron–hole pairs are generated and extracted from the quantum dot layer so that the electrons are transferred to the IGZO layer. For high photoresponsivity, it is important that a large number of electron–hole pairs are generated by the light source so that the majority carriers should be extracted from the QD layer, and transferred to the IGZO layer. While the minority carrier trapping may not be harmful or can often increase the lifetime of the opposite carriers, the loss of majority carriers can drastically affect the overall photocurrents^14^. For example, since the electrons, in the graphene-PbS QD hybrid phototransistors, act as minority carriers, the high density trap states of PbS QDs effectively capture the electrons so that the majority carriers, holes in this case, can contribute to the improvement of photoconductive gain by circulating through the graphene channel^15^. For high photoresponsivity in the case of n-type IGZO channel with n-type QDs, it is important that a large number of electron–hole pairs are generated and extracted from the QD layer so that the electrons can be transferred to the IGZO layer. During this extraction and transfer process, a major loss of photo-generated current may occur due to the process of majority carrier trapping. In particular, in hybrids with n-type IGZO, majority carrier (electron in this case) trap states on the surface of QDs and interfacial trap sites between QD and IGZO layers are a major viewpoint. Thus, the main challenges in hybrid phototransistors remain as follows: 1) reducing majority carrier trap states on QD surface and at the interface between QD and IGZO layer, and 2) improving the air stability of QDs to prevent aggregation and oxidation.

If one tries to passivate the surface of individual QDs with insulating materials, the unstable dangling bonds can effectively be terminated, reducing charge trapping and improving air-stability by preventing photo-oxidation. However, the insulating shell may not be ideal because it impedes the charge transfer from QDs to the active channel, which can even lower photocurrent generation. One approach to satisfy all the requirements is to passivate the pre-deposited QD layers with an additional infiltration layer through the ALD process. In previous research about QD-based photovoltaics or QD-LEDs, materials such as Al_2_O_3_ or ZnO were infiltrated into the QD layers using an ALD process to improve both the optoelectronic properties and stability of QDs, but there are few studies on the application of ALD in devices that hybridize QD with other materials such as oxide semiconductors^16,17^. Moreover, ALD has the advantage of being able to penetrate the closely packed QD layers with excellent uniformity and precise thickness control on an atomic scale. Therefore, the application of an infiltration layer in ALD is expected to improve both photoresponsivity and photostability of hybrid phototransistors.

In this work, we proposed an ALD infiltration method that can improve both the photoresponsivity and stability of micropatterned PbS QD/IGZO phototransistors. Lead sulfide (PbS) QDs (used as a light absorption layer) are effective materials for transferring the charge carriers to the IGZO layer by converting the optical signal in the near-infrared light into an electrical signal. The long native oleic acid (OA) ligands on PbS, which prevent the photogenerated charge from moving to the IGZO layer, were replaced with short tetrabutylammonium iodide (TBAI) ligands. In addition, we tried to passivate the surface trap states of PbS QD and the interface trap sites between PbS QD and IGZO by applying the infiltration process of Al_2_O_3_ through ALD. Even though the spacings between QDs are reduced by the short ligands, we confirmed that Al_2_O_3_ can permeate the QD layers and effectively fill the gap between QDs. As a result, the micro-patterned Al_2_O_3_ infilled PbS QD/IGZO hybrid phototransistor exhibited excellent photoresponsivity of 1.65 × 10^2^ A/W, a detectivity of 1.11 × 10^13^ Jones and a relaxation time of 0.03 s when illuminated by NIR light (880 nm).

## Results

**Figure 1:**
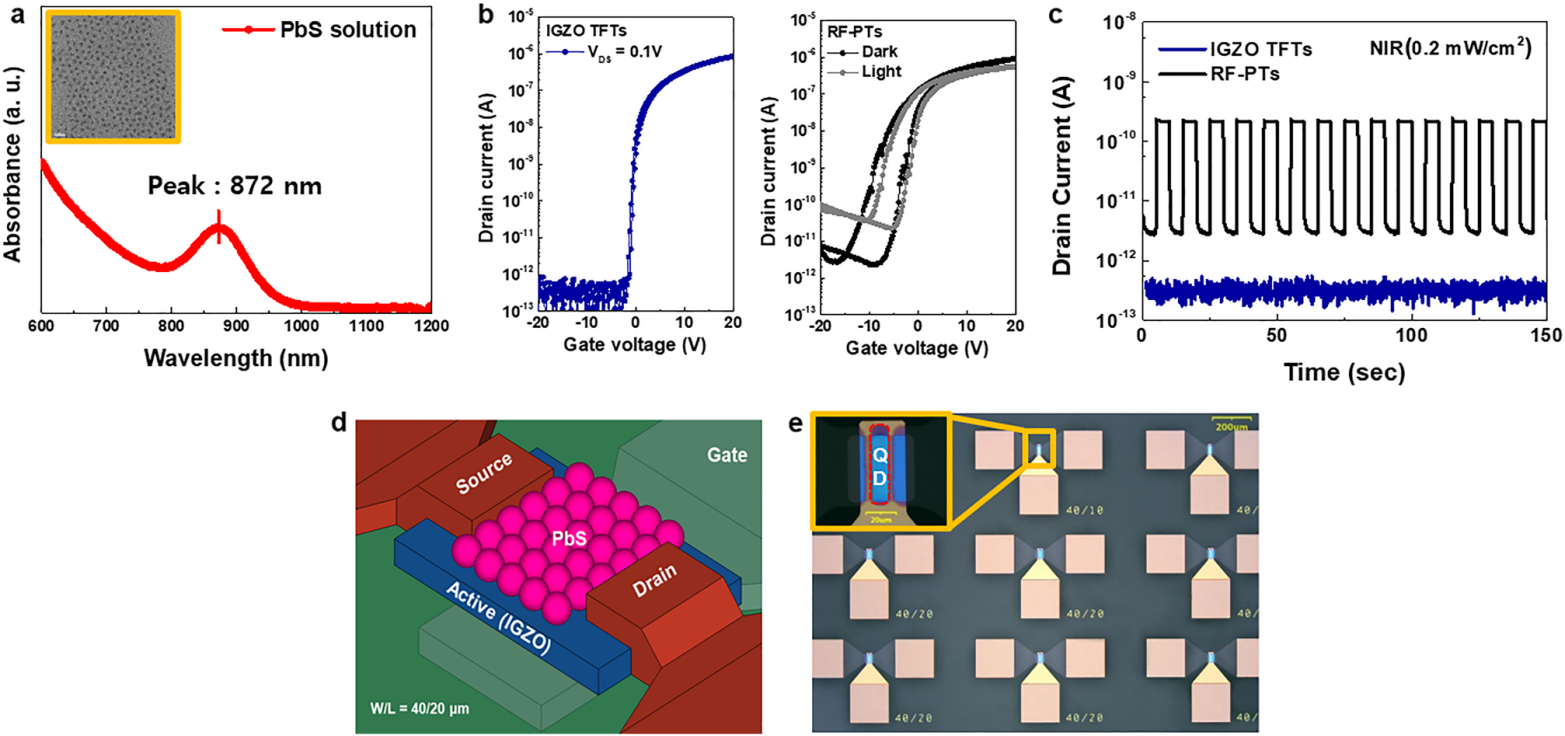
Device structure and the optoelectronic performance under NIR (880 nm, 0.2 mW/cm^2^). (**a**) The optical absorption spectra of PbS QDs. (**b**) Dark and photo-induced transfer curve of IGZO TFTs and RF-PTs. (**c**) Photoresponse under periodic illumination of IGZO TFTs and RF-PTs. (**d**) The three-dimensional (3D) structure scheme of RF-PTs. (**e**) The optical image of a micro-scale patterned phototransistor array.

PbS QD synthesis with high absorption peaks in the NIR and IGZO TFTs with stable characteristics were fabricated to construct a high-performance hybrid NIR phototransistor. Figure 1a shows the high absorption of synthesized PbS QDs in the near-infrared region of 872 nm. As shown in the inset of Fig. 1a, PbS QDs with a size of about 3 nm were uniformly synthesized to selectively absorb the near-infrared region of about 880 nm. The IGZO TFTs patterned by photolithography to have channel lengths of 40 μm and widths of 20 μm have stable operation as shown in Fig. 1b. They showed a linear mobility of 4.8 cm^2^/Vs, a threshold voltage of -0.98 V, and a subthreshold swing of 0.36 V/decade. The synthesized PbS QDs and micropatterned IGZO TFTs were hybridized by spin-coating QDs on the back-channel side of the coplanar bottom gate structure TFTs as shown in Fig. 1d. The native oleic acid (OA) ligands on PbS QDs were exchanged with tributylammonium iodide (TBAI) to improve charge transport properties between QDs and charge transfer from the QD active layer to the IGZO channel layer^18^. In the process of hybridization, the electrical properties such as hysteresis, threshold voltage, and mobility slightly deteriorated due to the adsorption of impurities such as organic matter on the IGZO backchannel in Fig. 1b^19,20^. However, even after hybridization, the switching characteristics of the device were maintained, and the photoresponse showed increased off-current without changing V_th_ or SS even under NIR light irradiation. Generally, the shift of V_th_ in the oxide-hybrid phototransistor is caused by the photo-gating effect and/or the increase of carrier concentration by the external light illumination^21^. However, we postulate that our devices would not be the case that the channels have a sufficient amount of photogenerated carriers, which is enough to cause the V_th_ shift. We rather consider that all the device structural parameters point in one possibility of the minimized photo-gating effect: the smaller area of the patterned QD layer than the active channel layer, and thicker PbS QD film. As a result, we can confirm that the hybrid phototransistors (RF-PTs) can be simultaneously applied as a switching transistor and a NIR photosensor. The stable photoresponse for a periodic light signal is also shown in Fig. 1c through the transient curve. Oxide semiconductors have the problem of persistent photocurrent (PPC), which is increased dark current under periodic light signals caused by ionized oxygen vacancies (V_o_^2+^) during the photoresponse^22^. The RF-PTs maintained a photocurrent and did not show a PPC phenomenon during repeated photoresponse. Since the IGZO TFTs did not react at all in the NIR region (Fig. 1c), the photoresponse in RF-PTs was entirely caused by PbS QDs. Therefore, we overcame the limitations of the oxide semiconductors as optical sensors including the PPC phenomena and large bandgap (> 3.0 eV) by conjugating PbS QDs. As shown in the schematic diagram in Fig. 1d, micro-scale photolithography patterning can be applied to the PbS QDs layer as well as the IGZO TFT through dry etching. Figure 1e shows an optical image of a phototransistor array that is actually patterned to a level of 10 µm. The inset shows a PbS QDs layer patterned up to 10 µm through dry etching. This strongly suggests that these hybrid phototransistors can be embedded within the micro pixel of the display and can act as a NIR sensor while at the same time serving as a switching transistor.

**Figure 2:**
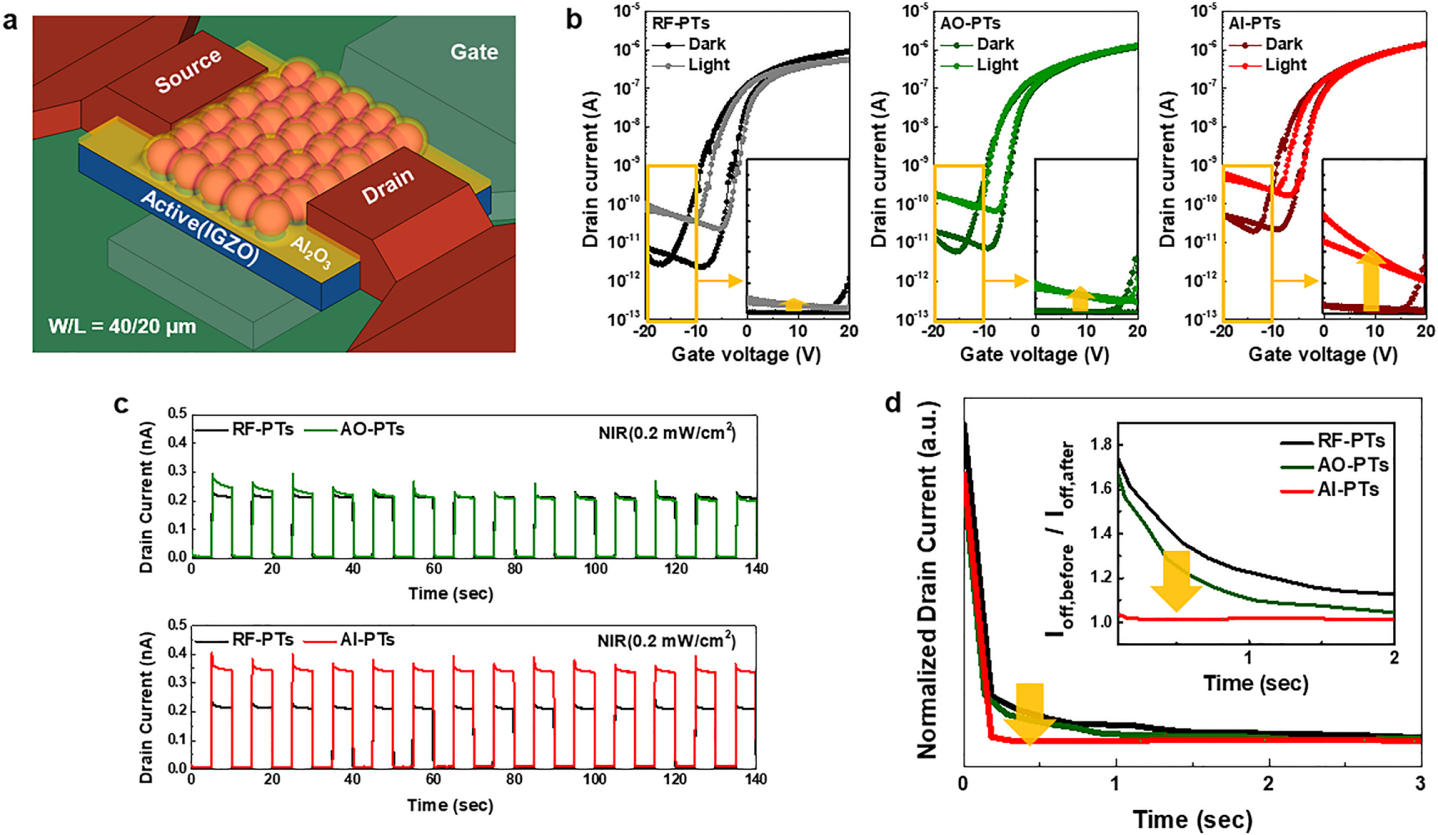
The phototransistor structure and the optoelectronic performance under NIR (880 nm, 0.2 mW/cm^2^). (**a**) The schematic three-dimensional (3D) view of AI-PTs. (**b**) Dark and photo-induced transfer curve of RF-PTs, AO-PTs and AI-PTs. (**c**) Transient curve under periodic illumination of RF-PTs, AO-PTs and AI-PTs. (**d**) A stretched exponential photo-relaxation of RF-PTs, AO-PTs and AI-PTs.

We additionally infilled the Al_2_O_3_ thin film to RF-PTs as shown in the schematic diagram of Fig. 2a to improve photoresponsivity, photo-relaxation and stability to external air. To confirm the effect of Al_2_O_3_ infiltration, the photoresponsivity and photo-relaxation properties of the following three devices were compared: 1) RF-PTs, 2) Al_2_O_3_ overcoated phototransistors (AO-PTs), and 3) Al_2_O_3_ infilled phototransistors (AI-PTs). The ALD of Al_2_O_3_ was just 14 cycles, which was sufficient for trap passivation^16^. Figure 2b–d show the electrical and optical properties of both AO-PTs that simply overcoat the Al_2_O_3_ without an exposure process and AI-PTs that infill the Al_2_O_3_ inside of PbS QDs with an exposure process. Both AO-PTs and Al-PTs enable simultaneous switching operations with an optical response that is the same as RF-PTs (Fig. 2b). Similarly, V_th_ does not move, and only an increase in off-current occurs. The improvement of photocurrent by Al_2_O_3_ is clearly shown as an increase in the linear scale of the off state in Fig. 2b inset. The micro-patterned thin QD layer may not provide enough carriers to negatively shift V_th_. On the other hand, even with such small carriers, a definite off-current change occurred due to the low off-current characteristics of the oxide semiconductor. The Al_2_O_3_ infiltration process improves photoresponsivity more effectively than simply overcoating the Al_2_O_3_ on the PbS QDs layer. It is expected that infiltrated Al_2_O_3_ could effectively passivate the trap sites at the surface of PbS QDs and even the interface of QD/IGZO. Therefore, in AI-PTs, the electrons generated in PbS QDs by NIR were not trapped, and more electrons were transferred to IGZO. The transient curve in Fig. 2c shows the photoresponse for a periodic light signal (λ = 880 nm, frequency = 0.1 Hz, power = 0.2 mW/cm^2^) at V_GS_ = − 15 V and V_DS_ = 0.1 V. All devices operated stably without a PPC phenomenon. The initial photocurrent of AO-PTs increased compared to RF-PTs, but gradually decreased as the photoresponse was repeated. After more than five signals, the photocurrent was equal to the amount of photocurrent generated by RF-PTs. This is because the accumulated illumination stress could regenerate the trap states that were not well passivated in the AO-PTs. On the other hand, AI-PTs generated a higher photocurrent than AO-PTs and maintain the current level.

The numerical optoelectronic characteristics extracted under NIR (880 nm) at V_GS_ = − 15 V and V_DS_ = 0.1 V are shown in Table 1. Photosensitivity (PS), photoresponsivity (PR), external quantum efficiency (EQE), and detectivity (D^*^) were evaluated by the following equations^23,24^:

$$ PS = \frac{{I_{ph} }}{{I_{dark} }} $$

$$ PR = \frac{{J_{ph} }}{P} $$

$$ EQE = \frac{{J_{ph} /q}}{P/hc} $$

$$ D^{*} = \frac{PR}{{\sqrt {2qJ_{dark} } }} $$

**Table 1:** The parameters of optoelectronic characteristics extracted under NIR (880 nm) at V_GS_ =  − 15 V and V_DS_ = 0.1 V (photosensitivity (PS), photoresponsivity (PR), external quantum efficiency (EQE), and detectivity (D^*^)).

	Photosensitivity	Photoresponsivity (A/W)	EQE (%)	Relaxation time (s)/rate (1/s)	Detectivity (Jones)
RF-PTs	79.5	96.4	68.3	0.52/1.92	1.09 $\times$ 10^13^
AO-PTs	50.7	1.20 $\times$ 10^2^	68.1	0.32/3.13	9.75 $\times$ 10^12^
AI-PTs	47.9	1.65 $\times$ 10^2^	1.17 $\times$ 10^2^	0.03/33.3	1.11 $\times$ 10^13^

Here, I_ph_ is the photocurrent (I_ph_ = I_light_ − I_dark_), J_ph_ is the photocurrent density, P is the light power density, PR is photoresponsivity, q is the electron charge, and I_dark_ is the current without illumination. As for the photosensitivity, which reflects the ratio of dark current (I_dark_) and photocurrent, AI-PTs and AO-PTs had lower values than RF-PTs because of their higher dark current. However, AI-PTs had the highest photoresponsivity and EQE of 1.65 × 10^2^ A/W and 1.17 × 10^2^%, respectively, due to having the highest photocurrent under illumination. That is, the conversion efficiency of light into electricity increased 1.5 times compared to RF-PTs by an Al_2_O_3_ infiltration process. In addition, detectivity (indicating the minimum degree of detected noise) had a high value of about 10^13^ Jones for all devices due to the low off-current characteristics of the oxide semiconductors.

The dark relaxation transient photocurrent of RF-PTs, AO-PTs, and AI-PTs are shown in Fig. 2d. The stretched exponential fitting evaluates the decay of photocurrent through the relaxation rate (Supplementary Fig. S1). Here, the decay of AI-PTs is relatively faster than both of AO-PTs and RF-PTs.

As shown in Table 1, the relaxation rate (1/τ) can be evaluated by fitting the decay curve to a stretched-exponential function as follows^25–27^:

$$ I_{DS} \left( t \right) = I_{DS} \left( 0 \right) \exp \left[ { - \left( {\frac{t}{\tau }} \right)^{\beta } } \right] $$

Here, I_DS_(0) is the photo current at the onset of relaxation, β is the stretching exponent which characterizes the nonhomogeneous system (0 < β < 1), and τ is the relaxation time constant. The extracted relaxation times for AI-PTs, AO-PTs and RF-PTs were 0.03 s, 0.32 s, and 0.52 s, respectively. Therefore, we expect that infilled Al_2_O_3_ penetrating to the QD/IGZO interface would both effectively passivate defect states and reduce the charge transfer barrier between QD and IGZO.

**Figure 3:**
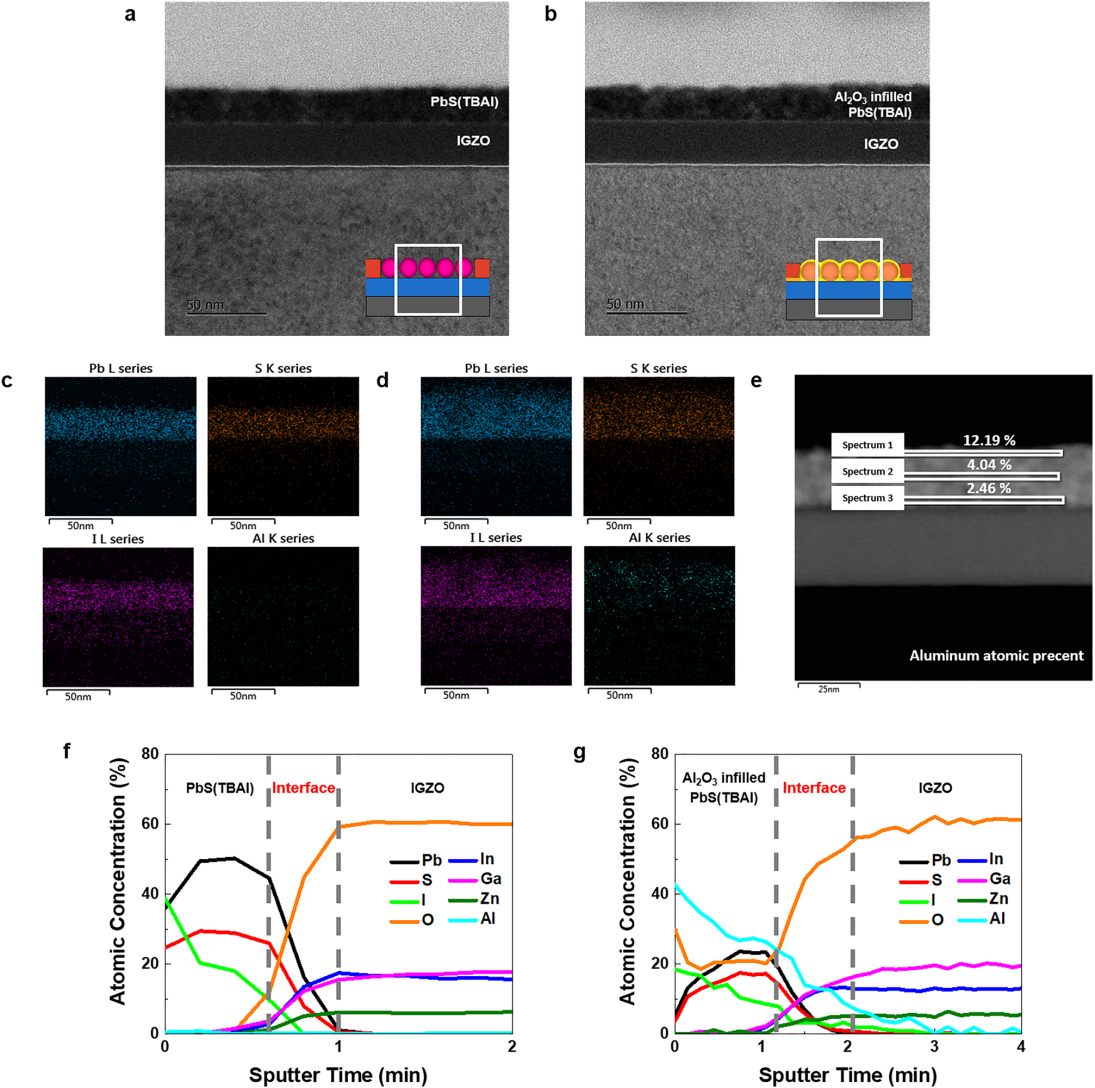
Confirm of the Infiltration Al_2_O_3_. Corresponding cross-sectional HRTEM images of (**a**) RF-PTs and (**b**) AI-PTs. EDS mapping images of (**c**) RF-PTs and (**d**) AI-PTs. (**e**) The vertical composition of aluminum in AI-PTs. AES concentration depth profile of (**f**) RF-PTs and (**g**) AI-PTs.

Just 14 cycles of Al_2_O_3_ ALD significantly improved the optical properties compared to RF-PTs. Although the same number of cycles (14) of Al_2_O_3_ were applied, the effect of Al_2_O_3_ was boosted through infiltration by adding exposure steps in the ALD sequence, resulting in remarkably high properties in both photoresponse and relaxation. Figure 3a–e shows the TEM analysis conducted to determine the penetration of Al_2_O_3_ and the effect on the PbS QDs layer in AI-PTs. The TEM cross-sectional images in Fig. 3a,b indicate that the uniform layer of the PbS QDs on the IGZO layer were about 20.32 ± 0.46 nm in thickness, and the morphology of the PbS layer did not change even after the Al_2_O_3_ infiltration process. Given that the average diameter of individual PbS QD is about 3 nm (Fig. 1a), we confirmed that the active layer consists of about 7–8 monolayers of PbS QDs. The thickness of PbS layers after Al_2_O_3_ ALD process slightly increased by about 2.4 nm, indicating thin Al_2_O_3_ layer is formed on the top of PbS layers. The EDS mapping in Fig. 3c,d shows the conformal distribution of iodine elements in the PbS QDs layer, which confirms the successful ligand exchange process from oleic acid (OA) to iodide ions. On the close-packed PbS QDs layer with short ligands, we infilled the Al_2_O_3_ through the ALD process. Figure 3b shows a cross-sectional SEM image of the infilled Al_2_O_3_, which is observed as bright spots in the PbS QDs layer. The elemental mapping of aluminum also demonstrates that the Al_2_O_3_ is entirely infilled at the PbS QDs layer. In addition, the vertical composition of aluminum is a gradual distribution of infilled Al_2_O_3_ from the top to the bottom of the PbS QDs layer as shown in Fig. 3e. Therefore, a single process of Al_2_O_3_ infiltration using the ALD technique could result in gradient deposition of Al_2_O_3_ in the PbS layer, which is appropriate for trap passivation without interfering with charge transfer.

The interface between photo absorption layer and charge transport layer is important for efficient photo current generation and photo relaxation. Since the trap passivation effect of Al_2_O_3_ at the interior of PbS was confirmed, we conducted an AES depth analysis in Fig. 3f,g to confirm whether Al_2_O_3_ penetrated to the interface between the PbS QD layer and the IGZO layer. In the case of infilling Al_2_O_3_, aluminum and oxygen were detected over the entire PbS QD thin film as same as the TEM-EDS results. In addition, a sufficiently large amount of aluminum is present at the interface between IGZO and QD. Therefore, since infilled Al_2_O_3_ penetrates even to the QD/IGZO interface, it plays a role in passivating trap sites at not only the entire QD layer but also the trap sites of the interface.

**Figure 4:**
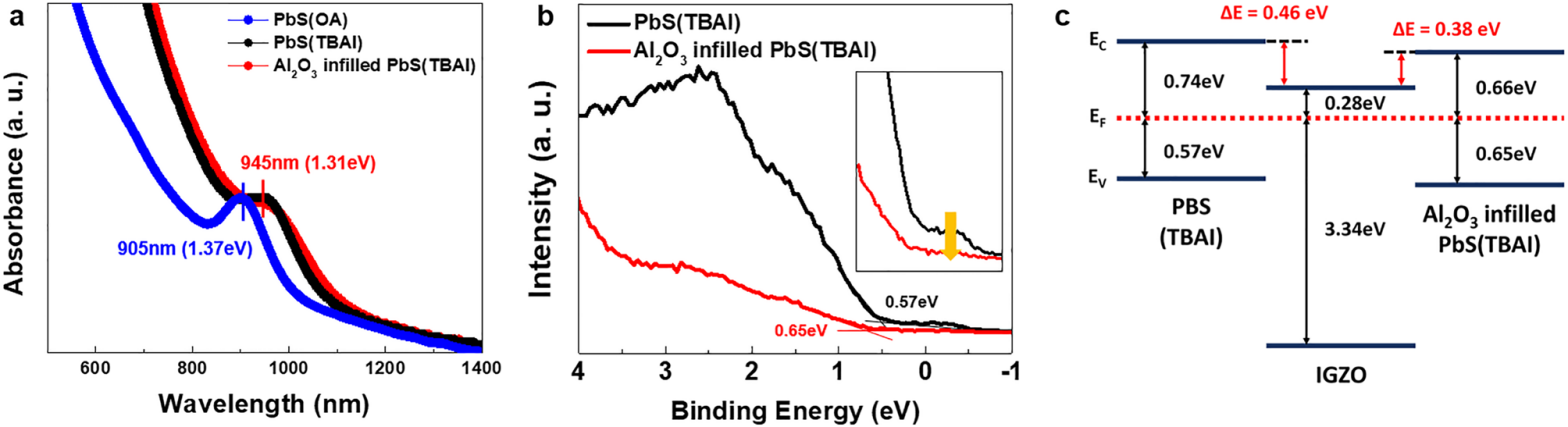
Optical analysis and energy band alignment. (**a**) Optical absorption spectra of PbS(OA), PbS(TBAI) and Al_2_O_3_ infilled PbS(TBAI). (**b**) The UPS spectra of PbS(TBAI) and Al_2_O_3_ infilled PbS(TBAI), showing relative defect states and fermi level energy. (**c**) Schematic energy band diagram of PbS(TBAI) and Al_2_O_3_ infilled PbS(TBAI) with IGZO.

The absorption spectra in Fig. 4a show the PbS QDs absorbance according to ligand and Al_2_O_3_ infiltration. The absorption of the PbS (TBAI) film increased compared to the PbS (OA) film as the first excitonic absorption peak red-shifted from 905 to 945 nm. By replacing the long chain OA ligand with the short TBAI ligand, the spacing between the quantum dot particles decreased, resulting in the red-shift due to slight aggregation of quantum dots^28^. Although the peak of Al_2_O_3_ infilled PbS (TBAI) slightly decreased, there was no shift in the position of the absorption pick compared to PbS (TBAI)^29^. Figure 4b and inset shows the different Fermi level positions and the defect states in the bandgap through UV photoelectron spectroscopy (UPS) analysis^30,31^. Here, the intersection of the tangents means the position of the valence band maximum, and the zero of the binding energy indicates the Fermi level. The higher intensity at the energy above the valence band maximum of PbS without Al_2_O_3_ represents that there are relatively more trap states in the bandgap compared to Al_2_O_3_ infilled PbS. As a result, the number of charge trap sites that decrease the carrier concentration in the PbS QD film is effectively passivated by infilling Al_2_O_3_ so that the Fermi level could be contributed to increase from 0.57 to 0.65eV^32,33^. When the band alignment of PbS and IGZO was determined (as shown in Fig. 4c), the conduction band offset (which acts as a charge transfer barrier) decreased from 0.46 to 0.38 eV due to an increase in Fermi level.

**Figure 5:**
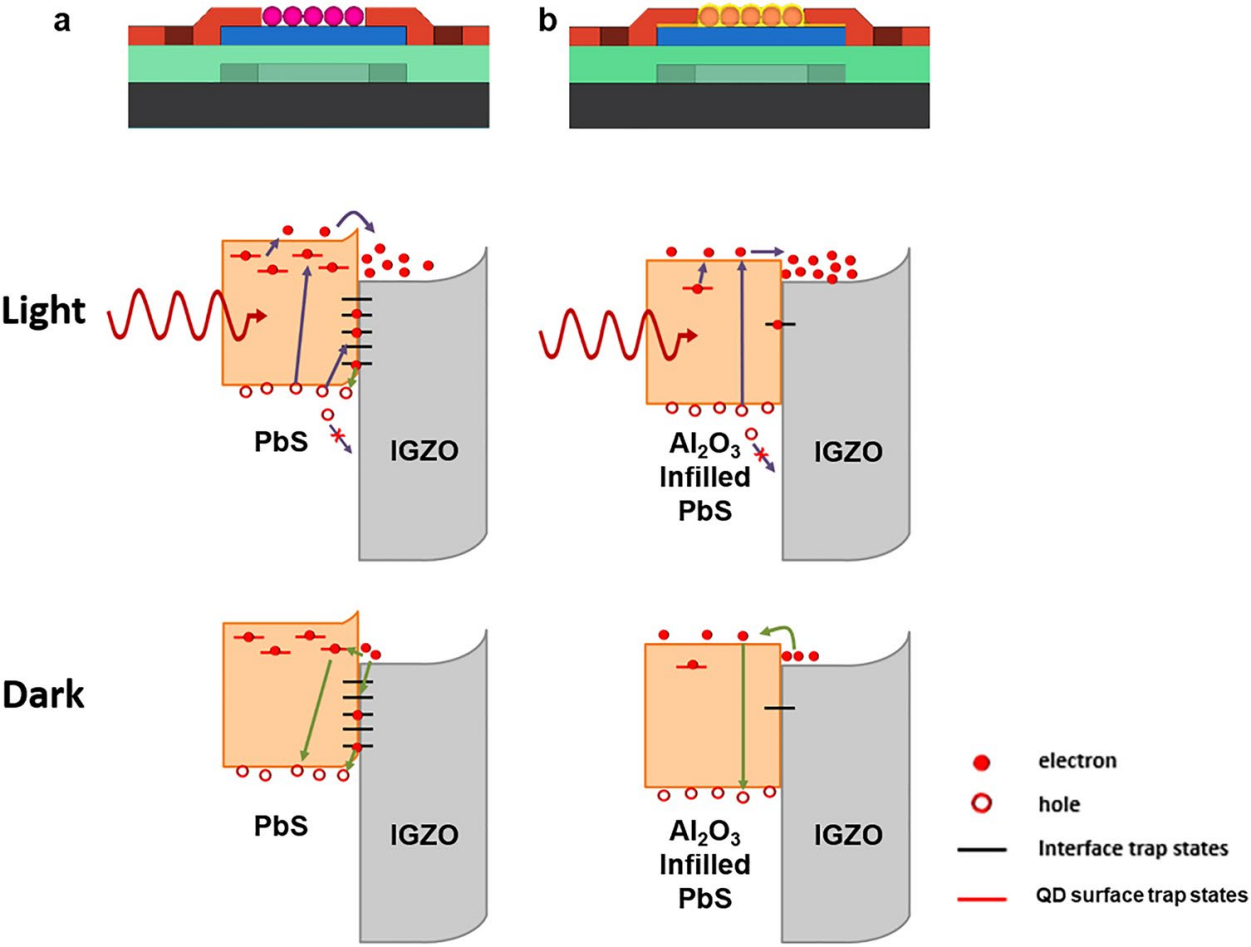
Operation mechanism. The phototransistor with and without illumination of (**a**) RF-PTs and (**b**) AI-PTs.

The underlying mechanisms governing the removal of defect states and the reduction of charge transfer barrier are shown in Fig. 5. These mechanisms can enhance the photocurrent and photo-relaxation behavior in Al_2_O_3_ infilled PbS(TBAI)/IGZO TFTs. First, the Al_2_O_3_ infiltration process effectively passivates QD surface trap states located within the band gap such as acceptor states and electron traps states. Previous studies suggest that only a thin Al_2_O_3_ layer can remove defect states (inevitably generated by dangling bonds and/or metal vacancies) of the QD surface and simultaneously can maintain relatively high charge transport properties in the close-packed QD solids^29,34,35^. Second, the optimized ALD process can help Al and O atoms (or ions) penetrate the QD layer and reach the IGZO/QD interface. The permeation of the precursors also reduced the surface defects on the back-channel of IGZO adjacent to QDs. It is well known that organic molecules such as ligands or solvent in QD solution may be able to affect the electrical properties of back-channel of IGZO during the process of depositing QDs on top of the IGZO layer. This undesirable back-channel effect in oxide semiconductor TFTs causes an increase in hysteresis^36^. The least hysteresis observed in AI-PTs revealed that even unavoidable IGZO surface defects during the QD layer formation can also be reduced by the Al_2_O_3_ penetration and infiltration process. Hosono's group showed that surface trap states in oxide semiconductors (which degrade optoelectronic properties such as reliability under illumination and exacerbate the PPC problem) can also be eliminated by depositing a passivation layer on the oxide semiconductors^37,38^. In addition, the reduction of QD/IGZO interface traps by the Al_2_O_3_ infilling process eliminates Fermi-level pinning, lowers the barriers at the interface^39,40^, and consequently improves the charge transfer at the heterojunction. Furthermore, the Al_2_O_3_ infiltration additionally lowers the transfer barrier between QDs and IGZO due to the relatively higher Fermi level of Al_2_O_3_ infilled PbS QDs. As a result, the infilling process of Al_2_O_3_ effectively improves both photocurrent and photo-relaxation rates by forming Al_2_O_3_ both on the PbS QDs surface and at the interface of the IGZO layer.

Finally, we evaluated the shelf stability improvement of infilled Al_2_O_3_ based on shelf time dependent normalized photosensitivity in Fig. S2. Although both RF-PTs and AI-PTs degraded similarly during the initial 2 weeks, the degradation of the Al_2_O_3_ infilled device to was largely stopped after 2 weeks, and it was stably maintained by saturation after 4 weeks. The effects of Al_2_O_3_ encapsulation properties have been demonstrated in many studies, and our results are consistent with previous studies on the increase in air stability by applying Al_2_O_3_ to PbS QDs^41,42^. Thus, gradually deposited Al_2_O_3_, which is about 2 nm on the top side of PbS QDs layer, effectively improved shelf stability by protecting PbS quantum dots from outside air.

## Discussion

In this work, we demonstrated Al_2_O_3_ infilled PbS/IGZO hybrid phototransistors via atomic layer deposition to improve the optoelectrical performance in the NIR (880 nm) region. All devices were micro-patterned with a channel length of 40 μm and a channel width of 20 μm to confirm the potential of a phototransistor array application. Using the ALD infiltration process, the Al_2_O_3_ deposited from top to bottom of the PbS QDs passivates both the surface trap sites of PbS QDs and the interface trap sites of PbS/IGZO. Moreover, this trap passivation by Al_2_O_3_ infiltration causes an increase in Fermi level and suppresses the Fermi level pinning effect of PbS QDs, which reduces the charge transfer barrier between PbS QDs and the IGZO layer. Therefore, AI-PTs have improved photoreactivity and photorecovery properties because relatively more photoexcited electrons are effectively transferred from PbS to IGZO than RF-PTs and AO-PTs. The AI-PTs showed improved photoresponsivity values of 1.65 $\times$ 10^2^ A/W, an EQE of 1.17 $\times$ 10^2^%, and a relaxation time of 0.03 s in the NIR region. In addition, the Al_2_O_3_ infiltration increased shelf stability since the Al_2_O_3_ is an effective encapsulation material that protects materials vulnerable to outside air. Based on the additional Al_2_O_3_ layer deposited by the ALD infiltration process, we offer an effective micro patterned quantum dot/metal oxide hybrid phototransistor with enhanced photoreactivity, photorecovery and air stability.

## Methods

### Preparation of PbS QDs

Oleic acid (OA, 90%), 1-octadecene (ODE, 90%), lead oxide (PbO, 99.999%), Bis(trimethylsilyl) sulfide ((TMS)_2_S, 95%), and hexane (95%, anhydrous) were purchased from Sigma-Aldrich. Ethanol (99.9%, anhydrous) was purchased from Daejung.

Synthesis of PbS QDs followed the previous method with slight modification. Briefly, 0.47 g of PbO, 23 ml of ODE, and 2 ml of OA were loaded in a 3-neck flask. The mixture was degassed at 110℃ for 1 h under vacuum. Then, the reaction flask was switched to N_2_ and heated up to 120℃. Once the PbO fully dissolved, the reaction flask was stabilized at 80℃. Meanwhile, a 21 μl (TMS)_2_S/1 ml pre-degassed ODE solution was prepared in a nitrogen-filled glove box. After removing the heating mantle, 5 ml of (TMS)_2_S precursor was swiftly injected into the reaction flask. The reaction flask was slowly cooled to room temperature. The as-synthesized PbS QDs solution was transferred into a glove box to prevent air exposure. Then, the solution was purified with hexane and ethanol twice. Finally, purified PbS QDs were redispersed in octane (50 mg/ml) and stored in a glove box until further use.

### Device fabrication

The IGZO TFTs with a bottom gate and top contact structure were fabricated on a glass substrate. A 100 nm thick aluminum oxide (Al_2_O_3_) buffer layer was grown at 200℃ via atomic layer deposition (ALD) using trimethylaluminum (TMA) and H_2_O. Then, a 100 nm thick molybdenum (Mo) gate electrode was deposited by direct current (DC) reactive sputtering. A 200 nm thick Al_2_O_3_ gate insulator was deposited on the gate electrode at 200℃ by ALD. The gate electrode and gate insulator layers were patterned using photolithography. A 30 nm thick indium gallium zinc oxide (IGZO) semiconductor layer was deposited by means of radio frequency (RF) sputtering with a plasma power of 100 W using an IGZO target (atomic ratio In:Ga:Zn = 1:1:1). A mixture of Ar and O_2_ gas (Ar/O_2_ = 9.7/0.3 sccm) was used for the deposition, and the working pressure was kept at 5 mTorr. After forming an IGZO layer, source/drain (S/D) electrodes were formed by RF sputtering 50 nm thick indium tin oxide (ITO). The active and S/D electrode layers were patterned by a lift-off process, and the channel width (W) and length (L) were 40 and 20 μm, respectively. The TFT devices were annealed at 450℃ in a low vacuum condition for 2 h.

The PbS QDs layers were capped with oleic acid (OA) ligands and were deposited by spin coating at 2500 rpm for 30 s. The entire ligand exchange process was carried out to replace the longer OA ligands of PbS QD with the shorter tetrabutylammonium iodide (TBAI) ligands as follows. TBAI solutions were dissolved in methanol with a concentration of 0.8 wt%. The coated PbS QD film was completely covered with TBAI solutions, held for 30 s, then dried by spin coating at 2500 rpm for 10 s. After rinsing twice with methanol, the film was finally baked at 50℃ for 5 min on a hot plate. The coated PbS QD film was micropatterned by dry etching. The dry etching parameters for the RF source power, bias power and the chamber pressure were set to 350 W, 150 W and 5 mTorr, respectively. Dry etching was performed with a Ar/Cl_2_ gas mixture ratio of 4/6.

The infiltration and overcoating processes were performed by ALD on the top of the TFTs as follows. Al_2_O_3_ was infilled at 100℃ at a working pressure of 0.3 Torr by ALD using trimethylaluminum (TMA) and H_2_O as the precursor and reactant, respectively. The complete sequence for Al_2_O_3_ infiltration consisted of a pulse of TMA for 0.2 s and an exposure of 1 s, a subsequent N_2_ purge of 20 s, followed by a H_2_O pulse of 0.3 s and an exposure of 1 s, and a final N_2_ purge of 30 s. During the exposure time (1 s) of TMA and H_2_O, the valve between the reaction chamber and the pump was closed. The growth rate and refractive index of Al_2_O_3_ were about 1.4 Å/cycle and 1.66, respectively, and we performed 14 cycles of Al_2_O_3_ infiltration process on the devices. The overcoating process was performed in the same way except for the previously mentioned exposure process.

### Material, electrical and photoelectric characterization

All electrical and photoresponse characteristics of the hybrid phototransistors were carried out using a semiconductor characterization system (Keithley 4200-SCS) in an air ambient atmosphere at room temperature. The photoelectric characteristics were measured under various laser sources with wavelengths of 700 nm (red), 880 nm (NIR) and intensities of 0.2 mW/cm^2^, and a transient response of the phototransistors was observed at frequencies of 0.1 Hz. The cross-section image of the QD/IGZO phototransistor and distribution of elements in QD were measured using a transmission electron microscope (TEM) (FE-TEM, JEM-2100F HR, JEOL). Ultraviolet photoelectron spectroscopy (UPS) (x-ray photoelectron spectroscopy-theta probe, installed at Hanyang Linc + Analytical Equipment Center) and ultraviolet–visible (UV–Vis) measurements (Shimadzu UV-2600) were conducted to analyze the band energy alignment of QD/IGZO films. Auger electron spectroscopy (AES) was carried out to confirm the infiltration of Al_2_O_3_ between the QD and IGZO layer.

## Supplementary Information

Supplementary Information.

## Data Availability

All datasets used and/or analyzed during the current study are available from the corresponding author upon reasonable request.
