# Supplementary material for: Enhanced performance and stability in InGaZnO NIR phototransistors with alumina-infilled quantum dot solid
Source: Sci Rep. 2022 Jul 16;12:12167. doi: 10.1038/s41598-022-16636-y (PMC9288469; doi:10.1038/s41598-022-16636-y)
Supplement: Supplementary file 1 — Supplementary Information. [file 41598_2022_16636_MOESM1_ESM.docx]

**Supporting Information**

**Enhanced Performance and Stability in InGaZnO NIR Phototransistors with Alumina-Infilled Quantum Dot Solid**

Yoon-Seo Kim^1^, Hye-Jin Oh^1^, Seungki Shin^1^, Nuri Oh^1*^ and Jin-Seong Park^1*^

^1^ Division of Materials Science and Engineering, Hanyang University, 222, Wangsimni-ro, Seongdong-gu, Seoul 04763, Republic of Korea

Correspondence: Professor JS Park, Division of Materials Science and Engineering, Hanyang University, 222, Wangsimni-ro, Seongdong-gu, Seoul 04763, Republic of Korea

**Corresponding Author**

^*^E-mail: [jsparklime@hanyang.ac.kr](mailto:jsparklime@hanyang.ac.kr) (Jin-Seong Park)

^*^E-mail: [irunho@hanyang.ac.kr](mailto:irunho@hanyang.ac.kr) (Nuri Oh)


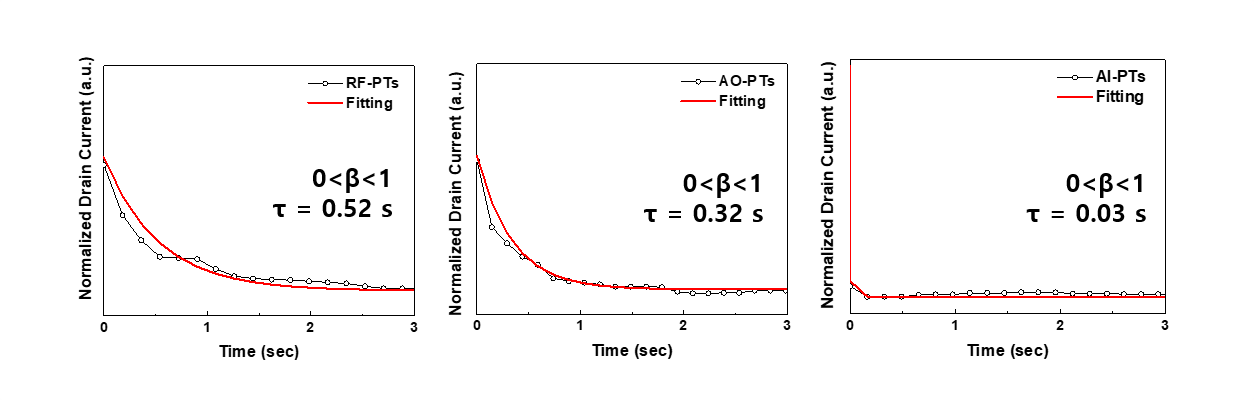


Fig. S1. Fits of stretched exponential photo-relaxation of RF-PTs, AO-PTs and AI-PTs.


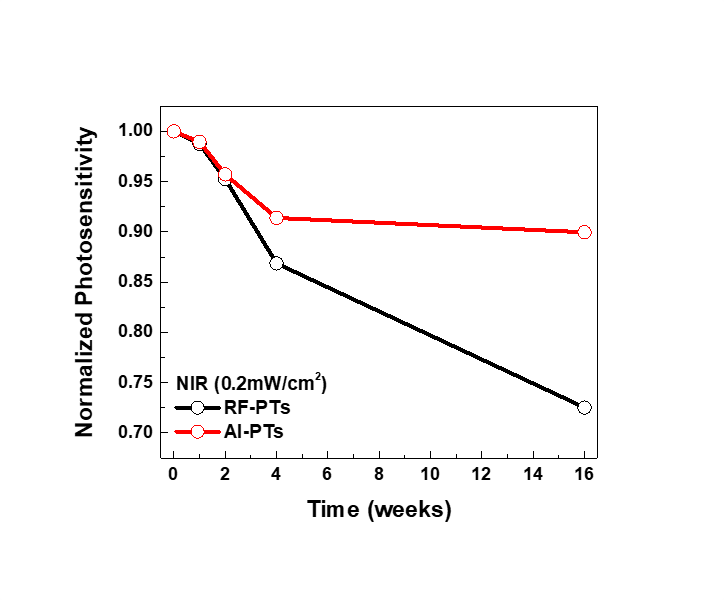


Fig. S2. Shelf stability of shelf time dependent normalized photosensitivity of (a) RF-PTs and (b) AI-PTs.
